# Science: the slow march of accumulating evidence

**DOI:** 10.1007/s40037-016-0305-1

**Published:** 2016-10-20

**Authors:** Katherine Picho, Lauren A. Maggio, Anthony R. Artino

**Affiliations:** Department of Medicine, Uniformed Services University of the Health Sciences, Bethesda, MD USA

**Keywords:** Replication, Reproducibility, Medical education

## Abstract

Recent crises over the credibility of research in psychology and the biomedical sciences have highlighted the need for researchers to view and treat replication research as essential to the accumulation of knowledge. In this article, the authors make the case for the utility of replication in medical education research. Specifically, the authors contend that because research in medical education often adopts theories from other disciplines, replication is necessary to gauge the applicability of those theories to the specific medical education context. This article introduces readers to the two major types of replication – direct and conceptual – and provides a primer on conceptual replication. In particular, the article presents key elements of conceptual replication and considers how it can be used to strengthen approaches to knowledge generation, theory testing, and theory development in medical education research.

Science is ever evolving, with new discoveries constantly pushing the boundaries of ‘what we know’ about how the world works. However, because randomness can influence what is observed about nature, there is always a chance that new findings, rather than being ‘true,’ are merely false positives. The possibility of obtaining chance findings can only be ruled out through rigorous research methods and by repeatedly recreating and testing the conditions that led to a particular observation or finding – a process known as replication.

True findings should, theoretically, be replicable. Thus, when results from a new discovery are reproduced in several repeated experiments, the findings provide a more or less robust empirical foundation for a theory [1]. Replication is central to scientific growth and theory development because it allows scientists to test and confirm core principles of working theories, or discover new, conflicting findings. In so doing, replication allows scientists to separate the proverbial wheat (true effects) from the chaff (false positives or findings based on chance).

Despite its importance for scientific discovery and theory development, replication has been relegated to a peripheral role within the research enterprise, which could be attributed to systems factors such as publication arrangements that prize innovation; or the unspoken, but all-too-real notion that non statistically significant results are not very interesting and are, thus, unlikely to be published. Therefore, the pressure to *publish or perish* leaves researchers with the decision of having to play by the rules inherent in the publication system or go out on a limb and attempt to publish a perhaps less marketable replication attempt. As a result, replication often takes a back seat and science ultimately suffers.

The credibility of scientific research has been challenged in recent years as efforts to demonstrate scientific legitimacy through replication have been less successful than expected [2, 3]. For example, attempts by the Reproducibility Project to replicate over 100 published psychology studies reported that 65 % of key findings were *not reproducible* [3]. These less-than-successful replication efforts are somewhat alarming and beg the question: Had replication efforts been more central to scientific research, would findings that once shaped a field have been refuted much earlier, perhaps changing the trajectory of subsequent research and extant theories?

Although previous work has highlighted the issue of replication in biomedical and psychology research, we believe that other fields, including medical education, are equally at risk of false positives and thus warrant similar scrutiny. In this article, we focus on the need for replication in medical education research. We introduce the two major types of replication – direct and conceptual – and describe their strengths and weaknesses. We also consider how conceptual replication, in particular, may strengthen approaches to knowledge generation in medical education.

## Replication: direct versus conceptual

Theory development requires discovery *and* replication. Robust theories stand the test of time and are gauged by how well they hold up when repeatedly tested under a wide array of conditions. Replication can have one of several outcomes: either the theory is confirmed, revised, extended, or rejected. Furthermore, replication can be thought of as either being direct or conceptual.

When considering replication, researchers may first think of direct replication in which a scientist endeavours to precisely replicate all elements of a study. However, replication can be conceived of as being either direct or conceptual. Direct replication refers to research intended to gauge the veracity of scientific findings by repeating, as closely as possible, an experimental procedure [4]. Conceptual replication, on the other hand, is an attempt to test the theory underlying a particular result [1].

Because direct replication focuses primarily on validating a specific finding from a particular study [1], a scientist conducting a direct replication study attempts to keep all conditions as similar to the original study as possible. Consider, for example, an original study on test-enhanced learning that seeks to investigate whether repeated testing improves retention of knowledge and hence future performance among medical residents [5]. In this study, residents participate in three one-hour teaching sessions that cover material that will be tested using multiple-choice questions. Participants are then randomly assigned to either a control or treatment group. The treatment group is repeatedly tested at 2, 4, 6, and 8 weeks on the topics covered, while the control group participates in a study session at 2, 4, 6, and 8 weeks and receives only one test at the end of 8 weeks. In a direct replication of this study, researchers might test a similar group of residents using the same methods and time frame. The researchers would not increase the length of the test, shorten the time in between tests, and change the testing format. In short, the direct replication study would attempt to match the procedures of the original study with as much fidelity as possible.

Direct replications are useful because they control for chance results or false positives [4, 6], and study artifacts (lack of internal validity) [4]. Although valuable, the role of direct replication in theory development is limited for a number of reasons. First, direct replications focus on replicating findings or confirming facts [4]. This focus on findings replication sheds little light on the credibility of the theory underlying a particular result, which consequently limits the role of direct replication in establishing generality [1]. Second, because direct replication requires that study conditions closely match those of the original study, any methodological or design flaws in the original study design (that might have contributed to a particular finding) will likely be perpetuated in subsequent direct replication studies of a given phenomenon. Therefore, in the case of design flaws, it is possible for false original findings to be confirmed through direct replication studies [1]. In this way, the ability of the literature to self-correct over time can be significantly impacted when design flaws in the original study are perpetuated in subsequent direct replication studies.

Conceptual replications, on the other hand, focus on validating the theory underlying a given result [4, 7]. Thus, conceptual replication is much like theory testing; that is, determining whether a given theory or hypothesis holds (or not) under a variety of conditions. For example, the test-enhanced learning effect referenced above has its roots in educational psychology but has been conceptually replicated in medical education. These replication studies have been conducted in a variety of settings (laboratories *vs*. classrooms), populations (adult *vs*. child learners) and content areas (word pairs *vs*. general content knowledge) to build a robust body of evidence for the theory.

Unlike direct replication, several elements of conceptual replications can be, and usually are, varied to gauge whether or not a given theory or hypothesis will hold under a variety of conditions, while at the same time holding ‘essential conditions’ constant. Essential conditions are dictated by the theory and constitute those conditions that must be in place for the phenomenon to occur. Conceptual replications only require that the essential conditions of the replication study closely match those in the original [8], while allowing for flexibility to vary other non-essential conditions of a study. For instance, a variety of measures, independent variables, methods, contexts and populations that differ from the original study can be used in conceptual replications to triangulate theoretical postulates that underlie a given result. Varying the aforementioned non-essential elements enables conceptual replication studies to rule out the possibility that observed findings could be due to demand characteristics, sample characteristics, or narrow definitions of a phenomenon [6].

Conceptual replication also facilitates falsification. Successful conceptual replications lend credence to extant theories, while unsuccessful ones (such as when a given theory does not operate as posited under certain conditions) might call for revisions to the bounds of said theory [3]. Because conceptual replications further our understanding of theory, we and others believe they are more valuable than their direct replication counterpart to theory development and, ultimately, to scientific progress [1].

One criticism of conceptual replication is that because there is flexibility in how dependent and independent variables are operationalized, as well as flexibility in how other methods are modified, when conceptual replications are unsuccessful, the source of replication failure is often difficult to ascertain [1]. The question becomes: Was the failure to replicate due to chance and/or artifacts, or was it due to other undetected moderators that might have influenced the results? For this reason, several scholars have suggested that direct replications should always precede conceptual replications [7–9], as verification of findings should precede extension of theory.

Direct and conceptual replications each serve different purposes, and so choosing which to conduct will depend largely on the researcher’s goal. If the goal is to verify a specific finding and ascertain that findings are less likely to be a result of chance, artifacts, or fraud, as well as other factors related to internal validity, then a direct replication is preferred. If the researcher’s goal is to extend or develop theory, then a series of temporally sequenced, hierarchically structured, and increasingly complex conceptual replication studies that help to progressively test and build robust theoretical frameworks is recommended (see Huffmeier et al. [8] for a detailed treatment of this issue).

## Replication in medical education

Although useful in domains such as cognitive psychology where experimental research prevails, direct replication is often impractical – and some might argue, irrelevant – in medical education contexts where factors associated with context specificity reign supreme. Additionally, direct replication’s contribution to theory development is limited because it emphasizes the validation of specific findings, which often sheds faint light on the credibility of the theory underlying a particular result [1]. Although direct replication may have limited use in medical education, we believe that conceptual replication has the potential to enhance the quality of medical education research, the methods of which have been criticized repeatedly over the past two decades [5]. Medical education scholars have argued that much of the research conducted in medical education was born out of convenience. Moreover, these critics have contended that the application of theory to medical education research is often ad hoc, with the primary intent being justification of an approach or explanation of findings rather than testing the tenets of a theory relative to the phenomenon in question [10]. Indeed, other scholars have proposed that medical education research can be improved by re-conceptualizing research quality as progressive accumulation of knowledge and advances in understanding of phenomena using methods that allow researchers to test theories and discard those that are weak and empirically unsupported [11]. Both theory development and results confirmation are facilitated by conceptual replication.

Further, medical education researchers often adopt theories from other disciplines including, but not limited to, education, psychology, and sociology. However, medical education researchers often do not ‘close the loop’ with respect to verifying whether or not the underlying theory applies to the specific context of medical education that was tested. Conceptual replication offers medical education researchers a means to test adopted theories within a medical education context and use subsequent findings to inform theory development, as well as practical applications in the field.

For medical education research to have a significant, positive impact on educational practice, we believe researchers must become fluent in the intricacies of replication – in particular, conceptual replication. There have been some positive, first steps toward this proposition. For example, policies at a few medical education journals, including this journal, explicitly solicit and encourage replication articles. However, there is still much work to be done with respect to attaining a critical mass of journals that openly promote the publication of replication studies. Additionally, it is notable that while some journals are calling for replication studies, we could find few examples of medical educators actually undertaking replication projects (with Larson et al. [7] being an exception). In the end, we believe that focused efforts aimed at promoting more replication have the potential to remedy false positives in our field and, in doing so, can help us build a more robust and relevant science of medical education.
